# The Y chromosome ancestry marker R1b1b2: a surrogate of the SARS-CoV-2 population affinity

**DOI:** 10.1038/s41439-021-00141-1

**Published:** 2021-02-18

**Authors:** Muntaser Ibrahim, Abdalhameed Salih

**Affiliations:** Institute of Endemic Diseases, University of Khartoum, Medical Campus Qasser Street, Khartoum, Sudan

**Keywords:** Diseases, Genetics

## Abstract

Individual and population susceptibilities to disease remain a murky area of investigation, clouded by past bias based on ideological differences and wars. The current SARS-CoV-2 pandemic, the largest in living memory, brought this matter to forefront as the disparity in disease burden became apparent. A timeline analysis of the pandemic revealed the presence of country clusters that display a marked preponderance of disease among populations carrying the ancestry marker R1b1b2, notably associated with both infection and mortality. This marker is a relic of past human expansions from western Asia and subsequently Europe and the rest of the world, which may have been accompanied by peculiar biological events rendering these populations vulnerable to SARS-CoV-2.

During epidemics, the human phenotypes of interest are infection, clinical disease, morbidity, and especially death. The current coronavirus disease (SARS-CoV-2) pandemic is associated with this range of phenotypes and the existence of marked human clusters of infection and particularly death. Populations, people within populations, and individuals obviously vary in their propensity to develop clinical symptoms, including death. In fact, epidemics without associated mortality are of little epidemiological value and in many cases may pass unnoticed. Populations are products of genetic history, that is, the history of a group’s genes upon interaction with various environments, including viral onslaughts and other environmental and genetic interactions^1^. There is also the history of the population itself, including migration, admixture, culture, and other non-biological determinants.

Genetic backgrounds pertain mainly to ancestry. When seeking markers of ancestry, the choice is usually either mitochondrial DNA or the Y chromosome, as they are both spared from the shuffling impact of recombination. However, given the unique history of males within the context of male-driven migration, they make a better marker of population structure and global human ancestry clusters^2–4^.

Populations as evolutionary units were rather cumbersome to define in the post-World War II era, in terms of both health and disease. In many cases, this was aggravated by a lingering tendency to adopt archaic terms for human groupings (Caucasoid, Mongoloid, and Negroid) mostly based on race myths. Entering the era of chronic and noninfectious diseases, also in the aftermath of World War II, heralded a departure from universal paradigms of health into one that accommodates human differences, currently best demonstrated in the arena of pharmacogenomics. Infectious diseases, however, remained aloof to these new approaches, as pathogens are widely believed to affect humans equally, despite the existence of human disease clusters, familial differences, and traits. The current SARS-CoV-2 pandemic raises several questions concerning the possible contribution of human genetic variation to the epidemiology of the disease and the extent to which it could explain the current disparities in disease burden across countries and communities.

Data from the current SARS-CoV-2 pandemic (www.worldmeters.info) for 95 countries as of 29 April, 31 May, 25 June, and 1 July 2020 (Supplementary tables: 1,2,3,4) were ranked by the number of deaths and the fatality rate using data derived from the World Meter Coronavirus update (www.worldmeters.info). Explaining complex phenomena such as the current pandemic in the biological and environmental dimensions is a cumbersome task and may often require equally complex analytical models. Regression analysis is perhaps the most convenient tool to capture the relative weight of multiple contributors to a complex phenomenon. As the main disease clusters included countries that are mainly in temperate and cold areas, suggesting temperature as an environmental factor potentially contributing to the epidemiology of the outbreak, multiple regression analysis of temperature readings against disease records by country from *World Bank Data Catalog* was carried out, and results are shown below. Given the current viral diversity and strain variations, neither temperature nor viral diversity seems to be able to adequately explain the current global trends in the pandemic. One of the foremost candidates naturally becomes the genetic background of the populations in the affected countries. To examine the relationship between ancestry and the clinical outcome of SARS-CoV-2, several ancestry markers were investigated, and the most prevalent and informative were included in subsequent analysis of populations affected by the pandemic. Haplotype frequencies were obtained from Y-chromosome haplogroup databases (https://en.wikipedia.org/wiki/Y-DNAhaplogroups_in_populations/http://thegeneticatlas.com/World_Y-DNA.htm) and the available literature^2,5–8^. For correlation and regression analysis, Python Software in the Statistical Package was used, Python Language Reference, version 3 (available at http://www.python.Org).

Among the 33 countries with the highest number of cases and deaths on 31 April (10,000 cases or more and an average death rate of 6.55% up to a staggering 16%, 23 are countries with populations that have a predominance of the Y chromosome haplotype R1b1b2 (M269)). The haplotype is believed to have originated in western Asia, specifically around the present area of Iran, which was coincidentally one of the primary foci for the first high fatality numbers in the pandemic. In the 33 countries recording between a thousand and 10,000 cases and a mortality index of 3.6, the R1b1b2 haplotype was still up to 40% in 22 countries, which was seen as a result of recent European expansion into the Americas and Northern Africa. Among thirty countries where case numbers were below a thousand and the mortality index was 3.1, most are in Africa, Asia, or South America, with lower frequencies of R1b1b2 that reach 0% in 11 countries. Overall, linear regression based on normal, Poisson, and polynomial distribution models for the correlation of R1b1b2 with both infection and mortality had the following values: 0.68 and 0.47, respectively. The coefficient for death to cases with R1b1b2 was 1.0927, *p* ≤ 0.001 when adjusted for country population size. Based on multiple linear regression, the coefficient for death to cases with temperature was −0.008, *p* ≤ 0.01 (Supplementary Fig. 1) and with R1b1b2 was 0.0359, with *p* ≤ 0.001 when adjusted for country population size (Figs. 1 and 2).

**Fig. 1:**
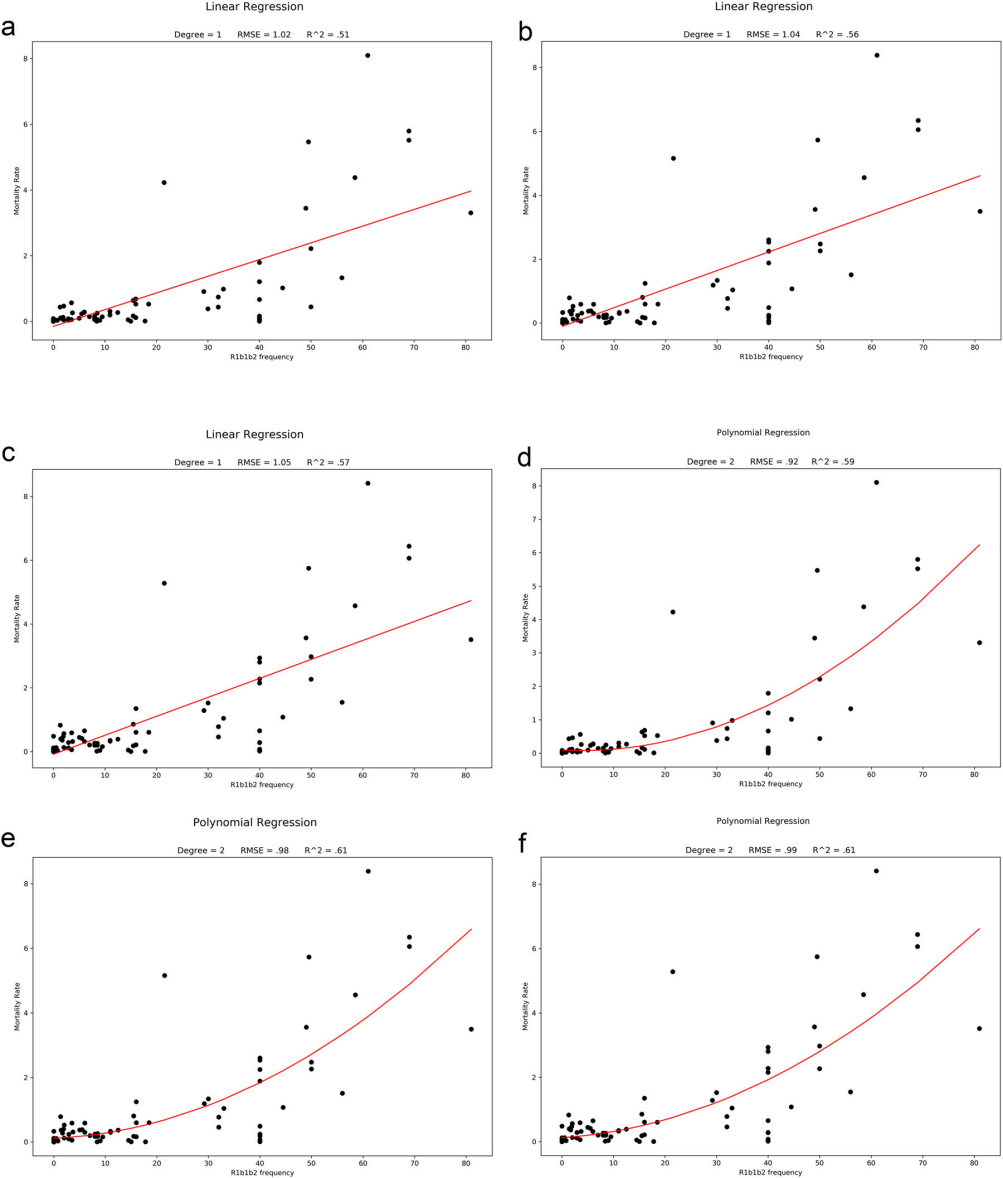
The correlation between the independent variable R1b1b2 and mortality/cases in the current SARS-CoV-2 pandemic as the dependent variable tested using different regression models on 31 May, 25 June and 1 July. : a, b and c are simple linear regression analyses that preliminarily gave an R^2 = 0.57, p ≤ 0.001 with a cluster at the bottom left of the figure depicting countries where the pandemic has a smaller impact in terms of mortality/cases. Better fitting was achieved with a regression polynomial distribution (d, e and f) (R^2 = 0.61, p ≤ 0.001).

**Fig. 2:**
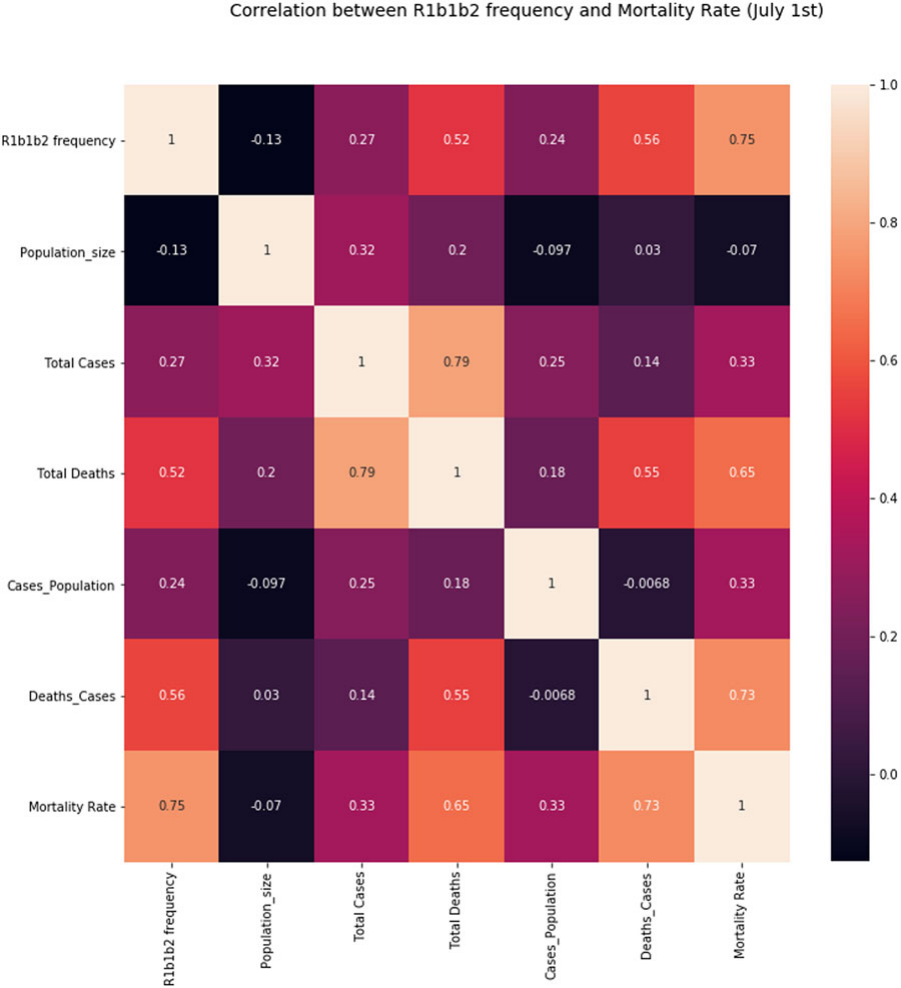
Correlation Heatmap. Matrix showing correlation between mortality rate, population size, total deaths, total cases, cases to population, deaths to cases, and frequency of R1b1b2 as of July first 2020.

The association with R1b1b2 is obviously noncausal, although recently collected evidence suggests that the Y chromosome influences immune and inflammatory responses in men, translating into genetically programmed susceptibility to diseases with a strong immune component^9^. However, the link in this analysis remains a tag for population history. Interestingly, such a tagging approach was reported for the risk of coronary artery disease by Charchar and his colleagues, who studied 3233 biologically unrelated British men to investigate heart disease risk; an odds ratio of 1.75 (95% CI 1.20–2.54, p ≤ 0.05) was reported between coronary artery disease and men carrying the haplotype R1b1b2^2^. We adopted a similar tagging haplotype approach earlier during the quest to understand the genetic basis of susceptibility to visceral leishmaniasis (VL) and to reveal hidden structures within seemingly homogenous populations^3^. A stratification of the target population based on Y chromosome haplogroups increased the likelihood of linkage to an impressive LOD score of 5.656, defining a susceptibility locus associated with carriers of haplogroup A1b1b2b formerly A-M13 A3b2; interestingly, this is the most common Y chromosome haplogroup among Nilotics of southern Sudan and other Nilo-Saharan speakers, who happen to be the most vulnerable population to VL in East Africa and worldwide^10^.

Population markers are not necessarily a reflection of ethnic identity. For example, African American, Caribbean and South American ethnic populations were found to carry substantial amounts of the R1b1b2 haplotype and its subclades. In some instances, the Y chromosome of Europeans has almost completely replaced the native male contribution to the gene pool^11^.

The events took place in time and space for ancestral carriers of R1b1b2 that rendered them vulnerable to SARS-CoV-2 infection and its severe pathology remain to be studied. Possible clues might be collected from the analysis of genomes/exomes of patients expressing the clinical outcome of such susceptibility loci. These loci might prove to be present at similar or higher frequencies in other populations that are phylogenetically divergent yet susceptible but show a high mortality phenotype, such as Indonesia and the Philippines, as outliers with no reported frequencies of R1b1b2. Even there, fatality was significantly lower when the ratio was corrected against the country population size. Similarly, in countries such as Japan and South Korea, mortality was 1.94 and 2.2, respectively, even without correction for the country population size. Interestingly, these ratios remained approximately the same, and the correlation with R1b1b2 even grew stronger over time when the same dataset was analyzed at the end of May, 25th of June, 1st of July and 18^th^ of November (Supplementary Figure 2). Among European countries, a linear positive correlation was found between R1b-S116 allele frequency and basic reproduction numbers. Evidently, disease burden does not vary only between continents, countries and regions in correlation with the average frequency of R1b1b2; even cities may become hot spots due to the unique history of human settlement, as noticed in northern Italy, where the epidemic is more intense than in southern Italy^12^.

The claim that the SARS-CoV-2 pandemic has yet to take its toll in Africa is largely speculative if not unfounded. Whether Africa’s population demographic pyramid, climate, degree of crowdedness, genetic background, natural or cross immunity and other confounders/contributors might have hindered or slowed the progression of SARS-CoV-2 remains to be studied. Anecdotes and forecasts of a pandemic on the loose with some countries peaking, and others waiting to strike, are embedded in a universal health paradigm that does not cater to human variations in disease susceptibility, a fact modern biomedical sciences is recognizing and embracing progressively and more than ever.

## Supplementary information

Supplementary Figure2

Supplementry Figure 1

Table
