# Supplementary figures and images for: The Y chromosome ancestry marker R1b1b2: a surrogate of the SARS-CoV-2 population affinity
Source: Hum Genome Var. 2021 Feb 18;8:11. doi: 10.1038/s41439-021-00141-1 (PMC7890103; doi:10.1038/s41439-021-00141-1)

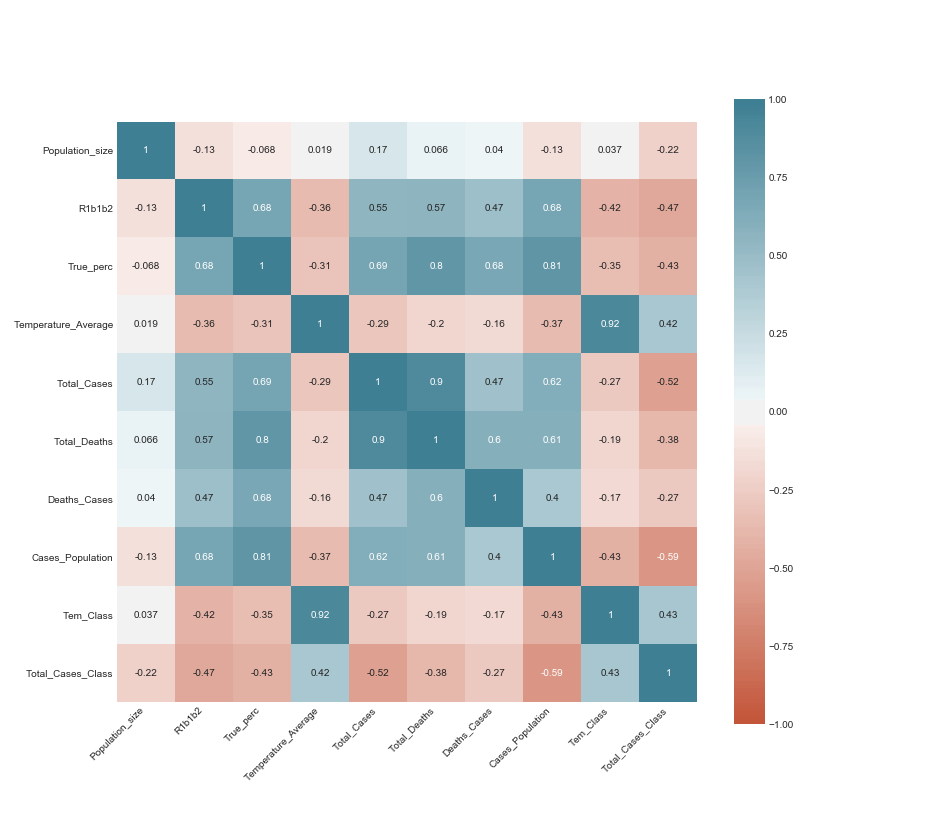

Supplement: Supplementary file 1 — Supplementary Figure2 [file 41439_2021_141_MOESM1_ESM.tif]

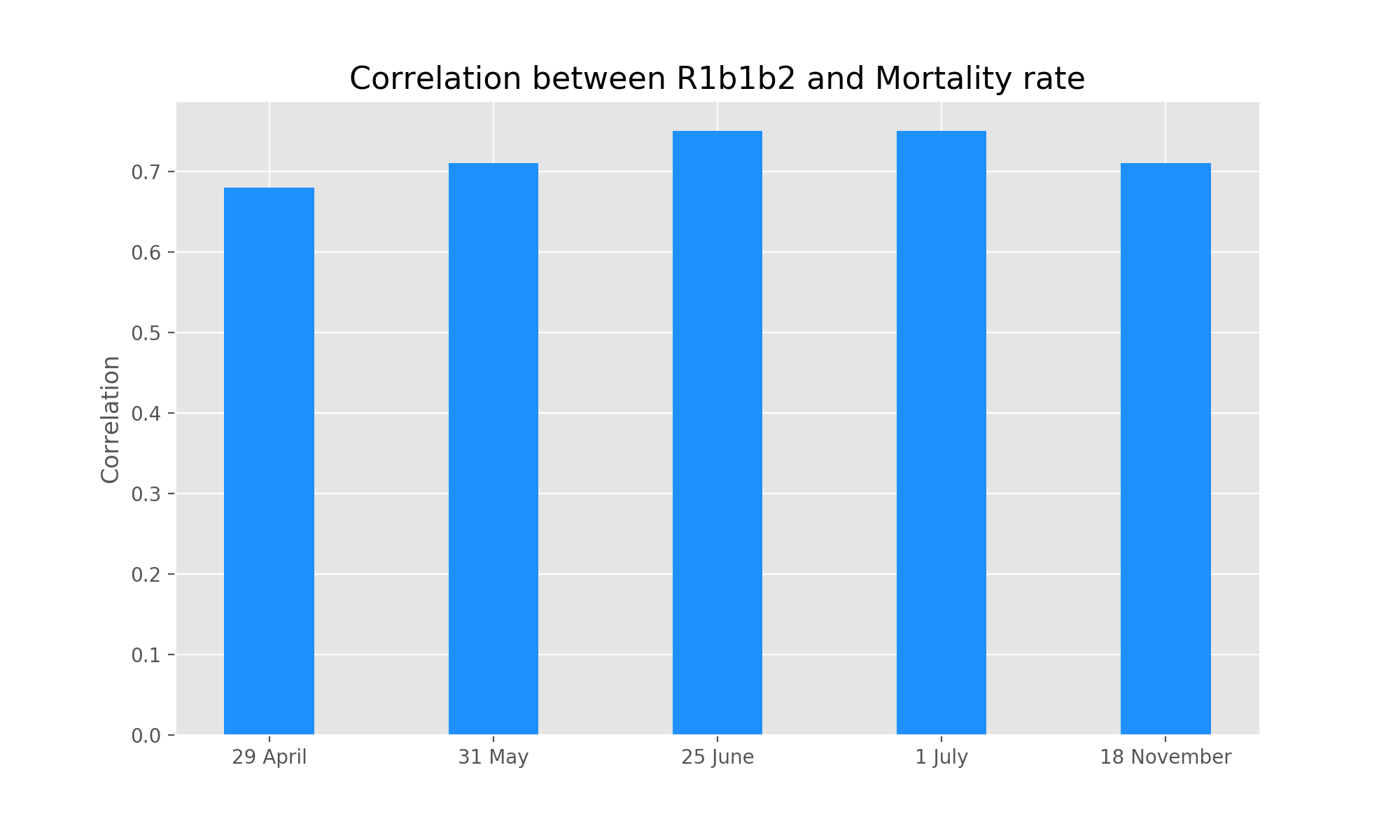

Supplement: Supplementary file 2 — Supplementry Figure 1 [file 41439_2021_141_MOESM2_ESM.tif]
