# Supplementary material for: The Y chromosome ancestry marker R1b1b2: a surrogate of the SARS-CoV-2 population affinity
Source: Hum Genome Var. 2021 Feb 18;8:11. doi: 10.1038/s41439-021-00141-1 (PMC7890103; doi:10.1038/s41439-021-00141-1)
Supplement: Supplementary file 3 — Table [file 41439_2021_141_MOESM3_ESM.docx]

**Supplementary table 1 April 29^th^ Deaths cases and R1b1 frequency by country**

| **Country** | **Population size** | **R1b** | **Total Cases** | **Total Deaths** | **Deaths/Cases** | **Cases/Population** | **Mortality** |
| --- | --- | --- | --- | --- | --- | --- | --- |
| Spain | 46754778 | 69.00 | **232128** | **23822** | 10.26244141 | 0.496479739 | 5.095094238 |
| Italy (North) | 60461826 | 49.50 | **199414** | **26977** | 13.52813744 | 0.329818024 | 4.461823565 |
| France | 65273511 | 58.50 | **165842** | **23293** | 14.04529613 | 0.254072437 | 3.568522612 |
| Germany | 83783942 | 44.50 | **158768** | **6136** | 3.864758642 | 0.189496932 | 0.732359907 |
| UK | 67886011 | 69.00 | **157149** | **21092** | 13.42165715 | 0.231489519 | 3.106972952 |
| Turkey | 84339067 | 16.00 | **112261** | **2900** | 2.583265782 | 0.133106761 | 0.34385014 |
| Russia | 145934462 | 6.00 | **93558** | **867** | 0.926697877 | 0.0641096 | 0.05941023 |
| Iran | 83992949 | 29.20 | **92584** | **5877** | 6.347749071 | 0.1102283 | 0.69970159 |
| China | 1439323776 | 0.80 | **82836** | **4633** | 5.592978898 | 0.005755203 | 0.032188727 |
| Brazil | 212559417 | 40.00 | **67446** | **4603** | 6.824719034 | 0.031730422 | 0.216551215 |
| Canada | 37742154 | 40.00 | **48500** | **2707** | 5.581443299 | 0.12850353 | 0.717235164 |
| Belgium | 11589623 | 61.00 | **47334** | **7331** | 15.48781003 | 0.408417081 | 6.32548617 |
| Netherlands | 17134872 | 49.00 | **38416** | **4566** | 11.88567264 | 0.224197765 | 2.664741236 |
| India | 1380004385 | 0.50 | **29451** | **939** | 3.188346745 | 0.002134124 | 0.006804326 |
| Switzerland | 8654622 | 50.00 | **29264** | **1677** | 5.730590487 | 0.338131463 | 1.937692946 |
| Peru | 32971854 | 40.00 | **28699** | **782** | 2.724833618 | 0.087040905 | 0.237171983 |
| Portugal | 10196709 | 56.00 | **24322** | **948** | 3.897705781 | 0.238527941 | 0.929711733 |
| Saudi Arabia | 34813871 | 1.90 | **20077** | **152** | 0.757085222 | 0.057669542 | 0.043660758 |
| Ireland | 4937786 | 81.00 | **19648** | **1102** | 5.608713355 | 0.397911129 | 2.231769461 |
| Sweden | 10099265 | 21.50 | **19621** | **2355** | 12.00244636 | 0.194281465 | 2.331852863 |
| Israel | 8655535 | 11.00 | **15589** | **208** | 1.334274168 | 0.180104407 | 0.240308658 |
| Mexico | 128932753 | 40.00 | **15529** | **1434** | 9.234335759 | 0.012044263 | 0.111220769 |
| Austria | 9006398 | 32.00 | **15357** | **569** | 3.705150746 | 0.170512118 | 0.631773102 |
| Pakistan | 220892340 | 8.50 | **14079** | **301** | 2.137935933 | 0.006373693 | 0.013626548 |
| Chile | 19116201 | 50.00 | **13813** | **198** | 1.433432274 | 0.072258081 | 0.103577065 |
| Japan | 126476461 | 0.00 | **13614** | **385** | 2.827971206 | 0.010764058 | 0.030440447 |
| Belarus | 9449323 | 5.50 | **12208** | **79** | 0.647116645 | 0.129194441 | 0.083603873 |
| Poland | 37846611 | 12.50 | **12089** | **570** | 4.715030193 | 0.031942094 | 0.150607937 |
| Qatar | 2881053 | 1.40 | **11921** | **10** | 0.08388558 | 0.413772326 | 0.034709532 |
| Romania | 19237691 | 15.50 | **11616** | **650** | 5.595730028 | 0.060381467 | 0.337878387 |
| UAE | 9890402 | 3.70 | **11380** | **89** | 0.782073814 | 0.115061046 | 0.089986231 |
| S. Korea | 51269185 | 0.00 | **10752** | **244** | 2.269345238 | 0.020971662 | 0.04759194 |
| Indonesia | 273523615 | 0.00 | **9511** | **773** | 8.127431395 | 0.003477213 | 0.028260814 |
| Ukraine | 43733762 | 8.00 | **9410** | **239** | 2.539851222 | 0.021516557 | 0.054648855 |
| Denmark | 5792202 | 33.00 | **8851** | **434** | 4.903400746 | 0.152808897 | 0.74928326 |
| Serbia | 8737371 | 6.00 | **8275** | **162** | 1.957703927 | 0.094708122 | 0.185410463 |
| Philippines | 109581078 | 0.00 | **7958** | **530** | 6.659964815 | 0.007262203 | 0.048366014 |
| Norway | 5421241 | 32.00 | **7599** | **206** | 2.710883011 | 0.140170858 | 0.379986796 |
| Bangladesh | 164689383 | 2.90 | **6462** | **155** | 2.398638193 | 0.00392375 | 0.009411657 |
| Malaysia | 32365999 | 0.00 | **5851** | **100** | 1.709109554 | 0.018077613 | 0.030896621 |
| Colombia | 50882891 | 40.00 | **5597** | **253** | 4.520278721 | 0.010999768 | 0.049722018 |
| South Africa | 59308690 | 5.00 | **4793** | **90** | 1.877738368 | 0.008081446 | 0.015174842 |
| Egypt | 102334404 | 2.90 | **4782** | **337** | 7.04726056 | 0.004672915 | 0.032931252 |
| Finland | 5540720 | 3.50 | **4740** | **193** | 4.071729958 | 0.085548449 | 0.348330181 |
| Morocco | 36910560 | 3.50 | **4246** | **163** | 3.838907207 | 0.011503483 | 0.044160804 |
| Argentina | 45195774 | 40.00 | **4003** | **197** | 4.921309018 | 0.008857023 | 0.043588146 |
| Algeria | 43851044 | 7.00 | **3517** | **432** | 12.28319591 | 0.008020334 | 0.098515328 |
| Moldova | 4033963 | 16.00 | **3481** | **102** | 2.930192473 | 0.086292314 | 0.252853088 |
| Kuwait | 4270571 | 1.30 | **3440** | **23** | 0.668604651 | 0.080551289 | 0.053856967 |
| Thailand | 69799978 | 0.00 | **2938** | **54** | 1.837985024 | 0.00420917 | 0.007736392 |
| Hungary | 9660351 | 18.50 | **2649** | **291** | 10.98527746 | 0.027421364 | 0.301231291 |
| Greece | 10423054 | 15.50 | **2534** | **136** | 5.367008682 | 0.024311493 | 0.130479992 |
| Oman | 5106626 | 1.70 | **2131** | **10** | 0.469263257 | 0.041730097 | 0.019582401 |
| Croatia | 4105267 | 8.50 | **2047** | **63** | 3.077674646 | 0.049862774 | 0.153461395 |
| Armenia | 2963243 | 30.00 | **1867** | **30** | 1.606855919 | 0.063005295 | 0.101240432 |
| Iraq | 40222493 | 0.00 | **1847** | **88** | 4.764482945 | 0.004591958 | 0.021878306 |
| Afghanistan | 38928346 | 8.00 | **1828** | **58** | 3.172866521 | 0.004695807 | 0.014899169 |
| Cameroon | 26545863 | 0.00 | **1705** | **58** | 3.401759531 | 0.006422846 | 0.021848979 |
| Bosnia and Herzegovina | 3280819 | 2.00 | **1585** | **63** | 3.974763407 | 0.048311108 | 0.192025223 |
| Ghana | 31072940 | 0.00 | **1550** | **11** | 0.709677419 | 0.004988263 | 0.003540058 |
| Cuba | 11326616 | 40.00 | **1389** | **56** | 4.031677466 | 0.012263151 | 0.049441069 |
| Bulgaria | 6948445 | 11.00 | **1387** | **58** | 4.181687094 | 0.019961301 | 0.083471914 |
| Slovakia | 5459642 | 14.50 | **1384** | **20** | 1.445086705 | 0.025349647 | 0.036632439 |
| Nigeria | 206139589 | 0.00 | **1337** | **40** | 2.991772625 | 0.00064859 | 0.001940433 |
| Guinea | 13132795 | 0.00 | **1163** | **7** | 0.60189166 | 0.008855693 | 0.005330168 |
| Tunisia | 11818619 | 0.33 | **967** | **39** | 4.033092037 | 0.008182005 | 0.032998779 |
| Senegal | 16743927 | 0.00 | **823** | **9** | 1.093560146 | 0.004915215 | 0.005375083 |
| Cyprus | 1207359 | 9.50 | **822** | **15** | 1.824817518 | 0.068082484 | 0.12423811 |
| Albania | 2877797 | 16.00 | **750** | **30** | 4 | 0.026061602 | 0.104246408 |
| Niger | 24206644 | 0.00 | **701** | **29** | 4.136947218 | 0.002895899 | 0.011980182 |
| Burkina Faso | 20903273 | 0.00 | **635** | **42** | 6.614173228 | 0.003037802 | 0.020092547 |
| Sri Lanka | 21413249 | 0.00 | **596** | **7** | 1.174496644 | 0.002783324 | 0.003269004 |
| Georgia | 3989167 | 9.00 | **511** | **6** | 1.174168297 | 0.012809692 | 0.015040734 |
| Somalia | 15893222 | 0.00 | **480** | **26** | 5.416666667 | 0.003020155 | 0.016359175 |
| DRC | 89561403 | 0.00 | **471** | **30** | 6.369426752 | 0.000525896 | 0.003349657 |
| Jordan | 10203134 | 17.80 | **449** | **7** | 1.559020045 | 0.004400609 | 0.006860637 |
| Mali | 20250833 | 0.00 | **408** | **23** | 5.637254902 | 0.002014732 | 0.011357557 |
| Kenya | 53771296 | 0.00 | **374** | **14** | 3.743315508 | 0.000695538 | 0.00260362 |
| Jamaica | 2961167 | 40.00 | **364** | **7** | 1.923076923 | 0.012292451 | 0.023639329 |
| El Salvador | 6486205 | 40.00 | **345** | **8** | 2.31884058 | 0.005318981 | 0.012333869 |
| Palestine | 5101414 | 8.40 | **342** | **2** | 0.584795322 | 0.006704024 | 0.003920482 |
| Venezuela | 28435940 | 40.00 | **329** | **10** | 3.039513678 | 0.001156987 | 0.003516676 |
| Sudan | 43849260 | 2.00 | **318** | **25** | 7.86163522 | 0.000725212 | 0.00570135 |
| Tanzania | 59734218 | 0.00 | **299** | **10** | 3.344481605 | 0.000500551 | 0.001674082 |
| Vietnam | 97338579 | 0.00 | **270** | **0** | 0 | 0.000277382 | 0 |
| Myanmar | 54409800 | 0.00 | **146** | **5** | 3.424657534 | 0.000268334 | 0.000918952 |
| Ethiopia | 114963588 | 0.00 | **126** | **3** | 2.380952381 | 0.0001096 | 0.000260952 |
| Cambodia | 16718965 | 0.00 | **122** | **0** | 0 | 0.00072971 | 0 |
| Zambia | 18383955 | 0.00 | **95** | **3** | 3.157894737 | 0.000516755 | 0.001631858 |
| Uganda | 45741007 | 0.00 | **79** | **0** | 0 | 0.000172712 | 0 |
| Libya | 6871292 | 0.00 | **61** | **2** | 3.278688525 | 0.000887752 | 0.002910661 |
| Chad | 16425864 | 0.00 | **46** | **0** | 0 | 0.000280046 | 0 |
| Syria | 17500658 | 15.00 | **43** | **3** | 6.976744186 | 0.000245705 | 0.001714221 |
| Mongolia | 3278290 | 0.00 | **38** | **0** | 0 | 0.001159141 | 0 |
| Zimbabwe | 14862924 | 0.00 | **32** | **4** | 12.5 | 0.000215301 | 0.00269126 |

**May 31**

| **Country** | **Population size** | **R1b** | **Total Cases** | **Total Deaths** | **Deaths Cases** | **Cases Population** | **Mortality** |
| --- | --- | --- | --- | --- | --- | --- | --- |
| Brazil | 212418030.00 | 40.00 | **414661.00** | **25697.00** | 6.20 | 0.20 | 1.21 |
| Russia | 145928826.00 | 6.00 | **379051.00** | **4142.00** | 1.09 | 0.26 | 0.28 |
| Spain | 46753147.00 | 69.00 | **283849.00** | **27118.00** | 9.55 | 0.61 | 5.80 |
| UK | 67852992.00 | 69.00 | **267240.00** | **37460.00** | 14.02 | 0.39 | 5.52 |
| Italy (North) | 60469746.00 | 49.50 | **231139.00** | **33072.00** | 14.31 | 0.38 | 5.47 |
| France | 65260368.00 | 58.50 | **182913.00** | **28596.00** | 15.63 | 0.28 | 4.38 |
| Germany | 83759426.00 | 44.50 | **181895.00** | **8533.00** | 4.69 | 0.22 | 1.02 |
| Turkey | 84252379.00 | 16.00 | **159797.00** | **4431.00** | 2.77 | 0.19 | 0.53 |
| India | 1378715135.00 | 0.50 | **159054.00** | **4541.00** | 2.86 | 0.01 | 0.03 |
| Iran | 83889077.00 | 29.20 | **143849.00** | **7627.00** | 5.30 | 0.17 | 0.91 |
| Peru | 32927196.00 | 40.00 | **135905.00** | **3983.00** | 2.93 | 0.41 | 1.21 |
| Canada | 37710897.00 | 40.00 | **87519.00** | **6765.00** | 7.73 | 0.23 | 1.79 |
| China | **1439323776.00** | 0.80 | **82995.00** | **4634.00** | 5.58 | 0.01 | 0.03 |
| Chile | 19100718.00 | 50.00 | **82289.00** | **841.00** | 1.02 | 0.43 | 0.44 |
| Saudi Arabia | 34760671.00 | 1.90 | **78541.00** | **425.00** | 0.54 | 0.23 | 0.12 |
| Mexico | 128803542.00 | 40.00 | **78023.00** | **8597.00** | 11.02 | 0.06 | 0.67 |
| Pakistan | 220462432.00 | 8.50 | **61227.00** | **1260.00** | 2.06 | 0.03 | 0.06 |
| Belgium | 11584977.00 | 61.00 | **57849.00** | **9388.00** | 16.23 | 0.50 | 8.10 |
| Qatar | 2876244.00 | 1.40 | **48947.00** | **30.00** | 0.06 | 1.70 | 0.10 |
| Netherlands | 17131422.00 | 49.00 | **45950.00** | **5903.00** | 12.85 | 0.27 | 3.45 |
| Bangladesh | 164533357.00 | 2.90 | **40321.00** | **559.00** | 1.39 | 0.02 | 0.03 |
| Belarus | 9449602.00 | 5.50 | **39858.00** | **219.00** | 0.55 | 0.42 | 0.23 |
| Sweden | 10093401.00 | 21.50 | **35727.00** | **4266.00** | 11.94 | 0.35 | 4.23 |
| UAE | 9878903.00 | 3.70 | **32532.00** | **258.00** | 0.79 | 0.33 | 0.26 |
| Portugal | 10199336.00 | 56.00 | **31292.00** | **1356.00** | 4.33 | 0.31 | 1.33 |
| Switzerland | 8648693.00 | 50.00 | **30796.00** | **1917.00** | 6.22 | 0.36 | 2.22 |
| South Africa | 59236519.00 | 5.00 | **25937.00** | **552.00** | 2.13 | 0.04 | 0.09 |
| Ireland | 4932505.00 | 81.00 | **24803.00** | **1631.00** | 6.58 | 0.50 | 3.31 |
| Indonesia | 273247624.00 | 0.00 | **24538.00** | **1496.00** | 6.10 | 0.01 | 0.05 |
| Kuwait | 4264401.00 | 1.30 | **24112.00** | **185.00** | 0.77 | 0.57 | 0.43 |
| Colombia | 50831115.00 | 40.00 | **24104.00** | **803.00** | 3.33 | 0.05 | 0.16 |
| Poland | 37850312.00 | 12.50 | **22600.00** | **1030.00** | 4.56 | 0.06 | 0.27 |
| Ukraine | 43756559.00 | 8.00 | **22382.00** | **669.00** | 2.99 | 0.05 | 0.15 |
| Egypt | 102141537.00 | 2.90 | **19666.00** | **816.00** | 4.15 | 0.02 | 0.08 |
| Romania | 19248782.00 | 15.50 | **18791.00** | **1229.00** | 6.54 | 0.10 | 0.64 |
| Israel | **9197590.00** | 11.00 | **16809.00** | **281.00** | 1.67 | 0.18 | 0.31 |
| Japan | 126510637.00 | 0.00 | **16651.00** | **858.00** | 5.15 | 0.01 | 0.07 |
| Austria | 9001628.00 | 32.00 | **16628.00** | **668.00** | 4.02 | 0.18 | 0.74 |
| Philippines | 109439758.00 | 0.00 | **15588.00** | **921.00** | 5.91 | 0.01 | 0.08 |
| Argentina | 45156510.00 | 40.00 | **13933.00** | **500.00** | 3.59 | 0.03 | 0.11 |
| Afghanistan | 38838960.00 | 8.00 | **13036.00** | **235.00** | 1.80 | 0.03 | 0.06 |
| Denmark | 5790332.00 | 33.00 | **11512.00** | **568.00** | 4.93 | 0.20 | 0.98 |
| S. Korea | 51265201.00 | 0.00 | **11344.00** | **269.00** | 2.37 | 0.02 | 0.05 |
| Serbia | 8740460.00 | 6.00 | **11275.00** | **240.00** | 2.13 | 0.13 | 0.27 |
| Oman | 5093172.00 | 1.70 | **9009.00** | **40.00** | 0.44 | 0.18 | 0.08 |
| Algeria | 43772270.00 | 7.00 | **8857.00** | **623.00** | 7.03 | 0.02 | 0.14 |
| Nigeria | 205612176.00 | 0.00 | **8733.00** | **254.00** | 2.91 | 0.00 | 0.01 |
| Norway | 5417258.00 | 32.00 | **8401.00** | **236.00** | 2.81 | 0.16 | 0.44 |
| Armenia | 2962740.00 | 30.00 | **8216.00** | **113.00** | 1.38 | 0.28 | 0.38 |
| Morocco | 36868513.00 | 3.50 | **7636.00** | **202.00** | 2.65 | 0.02 | 0.05 |
| Malaysia | 32325930.00 | 0.00 | **7629.00** | **115.00** | 1.51 | 0.02 | 0.04 |
| Moldova | 4034794.00 | 16.00 | **7537.00** | **276.00** | 3.66 | 0.19 | 0.68 |
| Ghana | 31007405.00 | 0.00 | **7303.00** | **34.00** | 0.47 | 0.02 | 0.01 |
| Finland | 5539940.00 | 3.50 | **6743.00** | **313.00** | 4.64 | 0.12 | 0.56 |
| Cameroon | 26477611.00 | 0.00 | **5436.00** | **175.00** | 3.22 | 0.02 | 0.07 |
| Iraq | 40130509.00 | 0.00 | **5135.00** | **175.00** | 3.41 | 0.01 | 0.04 |
| Sudan | 43744402.00 | 2.00 | **4346.00** | **195.00** | 4.49 | 0.01 | 0.04 |
| Hungary | 9662523.00 | 18.50 | **3816.00** | **509.00** | 13.34 | 0.04 | 0.53 |
| Senegal | 16697987.00 | 0.00 | **3348.00** | **39.00** | 1.16 | 0.02 | 0.02 |
| Guinea | 13095550.00 | 0.00 | **3275.00** | **20.00** | 0.61 | 0.03 | 0.02 |
| Thailand | 69784013.00 | 0.00 | **3065.00** | **57.00** | 1.86 | 0.00 | 0.01 |
| Greece | 10427500.00 | 15.50 | **2903.00** | **173.00** | 5.96 | 0.03 | 0.17 |
| DRC | 89271601.00 | 0.00 | **2660.00** | **69.00** | 2.59 | 0.00 | 0.01 |
| Bulgaria | 6952943.00 | 11.00 | **2477.00** | **134.00** | 5.41 | 0.04 | 0.19 |
| Bosnia and Herzegovina | 3282587.00 | 2.00 | **2462.00** | **153.00** | 6.21 | 0.08 | 0.47 |
| Croatia | 4107462.00 | 8.50 | **2245.00** | **102.00** | 4.54 | 0.05 | 0.25 |
| El Salvador | 6483178.00 | 40.00 | **2194.00** | **39.00** | 1.78 | 0.03 | 0.06 |
| Cuba | 11327235.00 | 40.00 | **1974.00** | **82.00** | 4.15 | 0.02 | 0.07 |
| Somalia | 15846666.00 | 0.00 | **1731.00** | **67.00** | 3.87 | 0.01 | 0.04 |
| Slovakia | 5459404.00 | 14.50 | **1520.00** | **28.00** | 1.84 | 0.03 | 0.05 |
| Sri Lanka | 21404986.00 | 0.00 | **1486.00** | **10.00** | 0.67 | 0.01 | 0.00 |
| Kenya | 53650862.00 | 0.00 | **1471.00** | **55.00** | 3.74 | 0.00 | 0.01 |
| Venezuela | 28443061.00 | 40.00 | **1245.00** | **11.00** | 0.88 | 0.00 | 0.00 |
| Mali | 20189289.00 | 0.00 | **1116.00** | **70.00** | 6.27 | 0.01 | 0.03 |
| Albania | 2878078.00 | 16.00 | **1076.00** | **33.00** | 3.07 | 0.04 | 0.11 |
| Tunisia | 11806826.00 | 0.33 | **1068.00** | **48.00** | 4.49 | 0.01 | 0.04 |
| Zambia | 18329866.00 | 0.00 | **1057.00** | **7.00** | 0.66 | 0.01 | 0.00 |
| Niger | 24110413.00 | 0.00 | **955.00** | **64.00** | 6.70 | 0.00 | 0.03 |
| Cyprus | 1206536.00 | 9.50 | **939.00** | **17.00** | 1.81 | 0.08 | 0.14 |
| Burkina Faso | 20843247.00 | 0.00 | **845.00** | **53.00** | 6.27 | 0.00 | 0.03 |
| Ethiopia | 114669653.00 | 0.00 | **831.00** | **7.00** | 0.84 | 0.00 | 0.00 |
| Georgia | 3989847.00 | 9.00 | **738.00** | **12.00** | 1.63 | 0.02 | 0.03 |
| Jordan | 10193504.00 | 17.80 | **720.00** | **9.00** | 1.25 | 0.01 | 0.01 |
| Chad | 16376161.00 | 0.00 | **715.00** | **64.00** | 8.95 | 0.00 | 0.04 |
| Jamaica | 2959976.00 | 40.00 | **569.00** | **9.00** | 1.58 | 0.02 | 0.03 |
| Tanzania | 59555005.00 | 0.00 | **509.00** | **21.00** | 4.13 | 0.00 | 0.00 |
| Palestine | 5089275.00 | 8.40 | **435.00** | **3.00** | 0.69 | 0.01 | 0.01 |
| Vietnam | 97255746.00 | 0.00 | **327.00** | **0.00** | 0.00 | 0.00 | 0.00 |
| Uganda | 45586273.00 | 0.00 | **281.00** | **0.00** | 0.00 | 0.00 | 0.00 |
| Myanmar | 54375747.00 | 0.00 | **206.00** | **6.00** | 2.91 | 0.00 | 0.00 |
| Mongolia | 3273095.00 | 0.00 | **161.00** | **0.00** | 0.00 | 0.00 | 0.00 |
| Zimbabwe | 14841813.00 | 0.00 | **132.00** | **4.00** | 3.03 | 0.00 | 0.00 |
| Cambodia | 16696479.00 | 0.00 | **124.00** | **0.00** | 0.00 | 0.00 | 0.00 |
| Syria | 17456890.00 | 15.00 | **121.00** | **4.00** | 3.31 | 0.00 | 0.00 |
| Libya | 6862224.00 | 0.00 | **99.00** | **4.00** | 4.04 | 0.00 | 0.01 |

**June 25^th^**

| **Country** | **Population size** | **R1b** | **Total Cases** | **Total Deaths** | **Deaths/Cases** | **Cases/Population** | **Mortality** |
| --- | --- | --- | --- | --- | --- | --- | --- |
| Brazil | 212533446 | 40.00 | **1,192,474** | **53,874** | 4.517834351 | 0.561075926 | 2.5348481 |
| Russia | 145933597 | 6.00 | **613,994** | **8,605** | 1.401479493 | 0.420735192 | 0.5896517 |
| India | 1379752264 | 0.50 | **474,272** | **14,914** | 3.144609001 | 0.034373707 | 0.1080919 |
| UK | 67880218 | 69.00 | **306,862** | **43,081** | 14.03920981 | 0.452063958 | 6.3466207 |
| Spain | 46754528 | 69.00 | **294,166** | **28,327** | 9.629596894 | 0.629171147 | 6.0586645 |
| Peru | 32962343 | 40.00 | **264,689** | **8,586** | 3.243806883 | 0.803004204 | 2.6047906 |
| Chile | 19113257 | 50.00 | **254,416** | **4,731** | 1.859552858 | 1.33109705 | 2.4752453 |
| Italy (North) | 60462972 | 49.50 | **239,410** | **34,644** | 14.47057349 | 0.39596135 | 5.7297878 |
| Iran | 83971321 | 29.20 | **212,501** | **9,996** | 4.703977864 | 0.253063781 | 1.1904064 |
| Mexico | 128907110 | 40.00 | **196,847** | **24,324** | 12.35680503 | 0.152704533 | 1.8869401 |
| Germany | 83779867 | 44.50 | **193,254** | **9,003** | 4.658635785 | 0.230668783 | 1.0746018 |
| Pakistan | 220791105 | 8.50 | **192,970** | **3,903** | 2.022594186 | 0.087399354 | 0.1767734 |
| Turkey | 84321768 | 16.00 | **191,657** | **5,025** | 2.621871364 | 0.227292435 | 0.5959315 |
| Saudi Arabia | 34802177 | 1.90 | **167,267** | **1,387** | 0.829213174 | 0.480622232 | 0.3985383 |
| France | 65271385 | 58.50 | **161,348** | **29,731** | 18.42663064 | 0.247195613 | 4.5549822 |
| Bangladesh | 164658783 | 2.90 | **122,660** | **1,582** | 1.289744008 | 0.074493445 | 0.0960775 |
| South Africa | 59293721 | 5.00 | **111,796** | **2,205** | 1.972342481 | 0.188546103 | 0.3718775 |
| Canada | 37736186 | 40.00 | **102,242** | **8,484** | 8.297959743 | 0.270938881 | 2.2482399 |
| Qatar | **2,807,805** | 1.40 | **90,778** | **104** | 0.114565203 | 3.233059276 | 0.3703961 |
| China | **1,439,323,776** | 0.80 | **83,449** | **4,634** | 5.553092308 | 0.005797792 | 0.0321957 |
| Colombia | 50872582 | 40.00 | **77,113** | **2,491** | 3.230324329 | 0.151580669 | 0.4896547 |
| Sweden | 10098211 | 21.50 | **62,324** | **5,209** | 8.357935948 | 0.617178627 | 5.1583394 |
| Belgium | 11588827 | 61.00 | **60,898** | **9,722** | 15.96439949 | 0.525488904 | 8.3891148 |
| Belarus | 9449365 | 5.50 | **59,945** | **362** | 0.603886896 | 0.634381252 | 0.3830945 |
| Egypt | 102289420 | 2.90 | **59,561** | **2,450** | 4.113429929 | 0.058227918 | 0.2395165 |
| Argentina | 45188207 | 40.00 | **49,851** | **1,116** | 2.23867124 | 0.110318606 | 0.2469671 |
| Netherlands | 17134314 | 49.00 | **49,804** | **6,097** | 12.2419886 | 0.290668188 | 3.5583566 |
| Indonesia | 273468761 | 0.00 | **49,009** | **2,573** | 5.250056112 | 0.017921243 | 0.0940875 |
| UAE | 9888042 | 3.70 | **46,133** | **307** | 0.665467236 | 0.466553439 | 0.310476 |
| Kuwait | 4269235 | 1.30 | **41,879** | **337** | 0.804699253 | 0.980948577 | 0.7893686 |
| Portugal | 10197071 | 56.00 | **40,104** | **1,543** | 3.847496509 | 0.393289406 | 1.5131796 |
| Ukraine | 43736564 | 8.00 | **40,008** | **1,067** | 2.666966607 | 0.09147495 | 0.2439606 |
| Iraq | 40199726 | 0.00 | **36,702** | **1,330** | 3.62378072 | 0.09129913 | 0.330848 |
| Oman | 5103139 | 1.70 | **33,536** | **142** | 0.423425573 | 0.657164149 | 0.2782601 |
| Poland | 37847153 | 12.50 | **32,821** | **1,396** | 4.253374364 | 0.086719865 | 0.3688521 |
| Philippines | 109551348 | 0.00 | **32,295** | **1,204** | 3.72813129 | 0.029479327 | 0.1099028 |
| Switzerland | 8653528 | 50.00 | **31,376** | **1,958** | 6.240438552 | 0.362580441 | 2.262661 |
| Afghanistan | 38906194 | 8.00 | **30,175** | **675** | 2.236951118 | 0.077558345 | 0.1734942 |
| Ireland | 4936723 | 81.00 | **25,396** | **1,726** | 6.796345881 | 0.514430321 | 3.4962464 |
| Romania | 19239018 | 15.50 | **24,826** | **1,555** | 6.263594619 | 0.12903985 | 0.8082533 |
| Armenia | 2963162 | 30.00 | **22,488** | **397** | 1.765385984 | 0.75891902 | 1.339785 |
| Israel | **9,197,590** | 11.00 | **22,139** | **308** | 1.391210082 | 0.240704358 | 0.3348703 |
| Nigeria | 206004225 | 0.00 | **22,020** | **542** | 2.461398728 | 0.010689101 | 0.0263101 |
| Japan | 126481147 | 0.00 | **18,024** | **963** | 5.342876165 | 0.014250345 | 0.0761378 |
| Austria | 9005551 | 32.00 | **17,449** | **693** | 3.971574302 | 0.193758272 | 0.7695254 |
| Moldova | 4034080 | 16.00 | **15,078** | **502** | 3.329354026 | 0.373765518 | 1.2443977 |
| Ghana | 31057126 | 0.00 | **15,013** | **95** | 0.63278492 | 0.048339953 | 0.0305888 |
| Serbia | 8737780 | 6.00 | **13,235** | **263** | 1.98715527 | 0.15146868 | 0.3009918 |
| Denmark | 5791888 | 33.00 | **12,615** | **603** | 4.780023781 | 0.217804626 | 1.0411113 |
| Cameroon | 26528317 | 0.00 | **12,592** | **313** | 2.48570521 | 0.04746626 | 0.1179871 |
| S. Korea | 51268565 | 0.00 | **12,563** | **282** | 2.244686779 | 0.024504294 | 0.0550045 |
| Algeria | 43832927 | 7.00 | **12,248** | **869** | 7.095035924 | 0.027942464 | 0.1982528 |
| Morocco | 36901974 | 3.50 | **10,907** | **216** | 1.980379573 | 0.029556684 | 0.0585335 |
| Sudan | 43822933 | 2.00 | **8,889** | **548** | 6.164922938 | 0.0202839 | 0.1250487 |
| Norway | 5420497 | 32.00 | **8,788** | **249** | 2.833409194 | 0.162125355 | 0.4593675 |
| Malaysia | 32357654 | 0.00 | **8,596** | **121** | 1.407631456 | 0.026565585 | 0.0373946 |
| Finland | 5540596 | 3.50 | **7,167** | **327** | 4.562578485 | 0.129354315 | 0.5901892 |
| DRC | 89480845 | 0.00 | **6,213** | **142** | 2.28553034 | 0.006943385 | 0.0158693 |
| Senegal | 16731859 | 0.00 | **6,129** | **93** | 1.517376407 | 0.036630717 | 0.0555826 |
| Kenya | 53741684 | 0.00 | **5,206** | **130** | 2.497118709 | 0.00968708 | 0.0241898 |
| Guinea | 13122901 | 0.00 | **5,174** | **29** | 0.560494782 | 0.039427258 | 0.0220988 |
| El Salvador | 6485677 | 40.00 | **5,150** | **119** | 2.310679612 | 0.079405743 | 0.1834812 |
| Ethiopia | 114888165 | 0.00 | **5,034** | **78** | 1.549463647 | 0.004381652 | 0.0067892 |
| Venezuela | 28436924 | 40.00 | **4,366** | **38** | 0.870361887 | 0.015353278 | 0.0133629 |
| Bulgaria | 6948964 | 11.00 | **4,242** | **209** | 4.926921264 | 0.061045071 | 0.3007643 |
| Hungary | 9660654 | 18.50 | **4,123** | **577** | 13.99466408 | 0.04267827 | 0.5972681 |
| Bosnia and Herzegovina | 3281035 | 2.00 | **3,676** | **173** | 4.706202394 | 0.112037817 | 0.5272726 |
| Greece | 10423625 | 15.50 | **3,310** | **190** | 5.740181269 | 0.031754788 | 0.1822782 |
| Thailand | 69797375 | 0.00 | **3,158** | **58** | 1.836605446 | 0.004524525 | 0.0083098 |
| Somalia | 15880718 | 0.00 | **2,835** | **90** | 3.174603175 | 0.017851838 | 0.0566725 |
| Croatia | 4105535 | 8.50 | **2,388** | **107** | 4.480737018 | 0.058165379 | 0.2606238 |
| Cuba | 11326708 | 40.00 | **2,319** | **85** | 3.665373006 | 0.020473733 | 0.0750439 |
| Albania | 2877838 | 16.00 | **2,114** | **47** | 2.223273415 | 0.073457922 | 0.163317 |
| Mali | 20234093 | 0.00 | **2,005** | **112** | 5.586034913 | 0.009909018 | 0.0553521 |
| Sri Lanka | 21411838 | 0.00 | **2,001** | **11** | 0.549725137 | 0.009345298 | 0.0051373 |
| Slovakia | 5459605 | 14.50 | **1,630** | **28** | 1.717791411 | 0.02985564 | 0.0512858 |
| Zambia | 18369406 | 0.00 | **1,489** | **18** | 1.20886501 | 0.008105869 | 0.0097989 |
| Palestine | 5098371 | 8.40 | **1,362** | **3** | 0.220264317 | 0.026714415 | 0.0058842 |
| Tunisia | 11816281 | 0.33 | **1,160** | **50** | 4.310344828 | 0.009816964 | 0.0423145 |
| Jordan | 10201247 | 17.80 | **1,071** | **9** | 0.840336134 | 0.010498716 | 0.0088225 |
| Niger | 24177857 | 0.00 | **1,051** | **67** | 6.374881066 | 0.004346953 | 0.0277113 |
| Cyprus | 1207207 | 9.50 | **991** | **19** | 1.917255298 | 0.082090313 | 0.1573881 |
| Burkina Faso | 20887260 | 0.00 | **919** | **53** | 5.767138194 | 0.004399811 | 0.0253743 |
| Georgia | 3989264 | 9.00 | **917** | **14** | 1.526717557 | 0.022986696 | 0.0350942 |
| Chad | 16412365 | 0.00 | **860** | **74** | 8.604651163 | 0.005239952 | 0.045088 |
| Uganda | 45697318 | 0.00 | **805** | **0** | 0 | 0.001761591 | 0 |
| Jamaica | 2960963 | 40.00 | **678** | **10** | 1.474926254 | 0.022897956 | 0.0337728 |
| Libya | 6869373 | 0.00 | **670** | **18** | 2.686567164 | 0.009753437 | 0.0262033 |
| Zimbabwe | 14858373 | 0.00 | **530** | **6** | 1.132075472 | 0.003567012 | 0.0040381 |
| Tanzania | 59685689 | 0.00 | **509** | **21** | 4.125736739 | 0.000852801 | 0.0035184 |
| Vietnam | 97322679 | 0.00 | **352** |  | 0 | 0.000361683 | 0 |
| Myanmar | 54403606 | 0.00 | **293** | **6** | 2.04778157 | 0.000538567 | 0.0011029 |
| Syria | 17489508 | 15.00 | **242** | **7** | 2.892561983 | 0.001383687 | 0.0040024 |
| Mongolia | 3277137 | 0.00 | **216** |  | 0 | 0.006591119 | 0 |
| Cambodia | 16714185 | 0.00 | **130** |  | 0 | 0.000777782 | 0 |

**July 1^st^**

| **Country** | **R1b** | **Total Cases** | **Total Deaths** | **Population** | **Cases/Population** | **Deaths/Cases** | **Mortality** |
| --- | --- | --- | --- | --- | --- | --- | --- |
| Brazil | 40.00 | **1,408,485** | **59,656** | 212558178 | 0.662635055 | 4.235472866 | 2.806572796 |
| Russia | 6.00 | **654,405** | **9,536** | 145934619 | 0.448423414 | 1.45720158 | 0.653443307 |
| India | 0.50 | **586,956** | **17,417** | 1379974505 | 0.042533829 | 2.967343378 | 0.126212477 |
| UK | 69.00 | **312,654** | **43,730** | 67886052 | 0.460557052 | 13.98670735 | 6.441676708 |
| Spain | 69.00 | **296,351** | **28,355** | 46754824 | 0.633840478 | 9.568046 | 6.064614851 |
| Peru | 40.00 | **285,213** | **9,677** | 32969875 | 0.865071524 | 3.392902848 | 2.935103636 |
| Chile | 50.00 | **279,393** | **5,688** | 19115944 | 1.461570509 | 2.035841986 | 2.975526608 |
| Italy (North) | 49.50 | **240,578** | **34,767** | 60461520 | 0.397902666 | 14.4514461 | 5.750268931 |
| Iran | 29.20 | **227,662** | **10,817** | 83988944 | 0.271061867 | 4.751341902 | 1.287907608 |
| Mexico | 40.00 | **226,089** | **27,769** | 128929303 | 0.175358894 | 12.28233129 | 2.153816034 |
| Pakistan | 8.50 | **213,470** | **4,395** | 220861534 | 0.096653318 | 2.058837307 | 0.198993456 |
| Turkey | 16.00 | **199,906** | **5,131** | 84336637 | 0.237033402 | 2.566706352 | 0.608395139 |
| Germany | 44.50 | **195,832** | **9,052** | 83784248 | 0.233733673 | 4.622329344 | 1.080394014 |
| Saudi Arabia | 1.90 | **190,823** | **1,649** | 34811071 | 0.548167564 | 0.864151596 | 0.473699876 |
| France | 58.50 | **164,801** | **29,843** | 65273746 | 0.2524767 | 18.10850662 | 4.571975998 |
| South Africa | 5.00 | **151,209** | **2,657** | 59305978 | 0.254964179 | 1.757170539 | 0.448015544 |
| Bangladesh | 2.90 | **149,258** | **1,888** | 164685660 | 0.090632056 | 1.264923823 | 0.114642647 |
| Canada | 40.00 | **104,204** | **8,591** | 37741604 | 0.276098493 | 8.244405205 | 2.276267855 |
| Colombia | 40.00 | **97,846** | **3,334** | 50881467 | 0.192301845 | 3.407395295 | 0.655248403 |
| Qatar | 1.40 | **96,088** | **113** | **2,807,805** | 3.422174973 | 0.117600533 | 0.4024496 |
| China | 0.80 | **83,534** | **4,634** | **1,439,323,776** | 0.005803698 | 5.54744176 | 0.032195675 |
| Sweden | 21.50 | **68,451** | **5,333** | 10099241 | 0.677783608 | 7.790974566 | 5.280594849 |
| Egypt | 2.90 | **68,311** | **2,953** | 102321109 | 0.066761395 | 4.322876257 | 0.28860125 |
| Argentina | 40.00 | **64,530** | **1,307** | 45194999 | 0.142781284 | 2.025414536 | 0.289191289 |
| Belarus | 5.50 | **62,118** | **392** | 9449314 | 0.657381054 | 0.631057021 | 0.414844929 |
| Belgium | 61.00 | **61,509** | **9,754** | 11589652 | 0.530723442 | 15.85784194 | 8.416128457 |
| Indonesia | 0.00 | **57,770** | **2,934** | 273516148 | 0.021121239 | 5.078760602 | 0.107269718 |
| Netherlands | 49.00 | **50,273** | **6,113** | 17134934 | 0.293394769 | 12.15960854 | 3.567565536 |
| Iraq | 0.00 | **49,109** | **1,943** | 40214559 | 0.122117465 | 3.956504918 | 0.483158351 |
| UAE | 3.70 | **48,667** | **315** | 9890001 | 0.492082862 | 0.647255841 | 0.318503507 |
| Kuwait | 1.30 | **46,195** | **354** | 4270271 | 1.081781461 | 0.766316701 | 0.8289872 |
| Ukraine | 8.00 | **44,998** | **1,173** | 43732279 | 0.102894249 | 2.606782524 | 0.26822293 |
| Portugal | 56.00 | **42,141** | **1,576** | 10196586 | 0.413285388 | 3.739825823 | 1.545615366 |
| Oman | 1.70 | **41,194** | **185** | 5105275 | 0.806890912 | 0.449094528 | 0.362370293 |
| Philippines | 0.00 | **38,511** | **1,270** | 109575261 | 0.035145707 | 3.297759082 | 0.115902074 |
| Poland | 12.50 | **34,775** | **1,477** | 37846476 | 0.09188438 | 4.247304098 | 0.390260906 |
| Afghanistan | 8.00 | **31,836** | **774** | 38920601 | 0.081797298 | 2.431209951 | 0.198866405 |
| Switzerland | 50.00 | **31,714** | **1,963** | 8654564 | 0.366442492 | 6.189695403 | 2.268167409 |
| Romania | 15.50 | **26,970** | **1,651** | 19236926 | 0.140199115 | 6.121616611 | 0.858245231 |
| Armenia | 30.00 | **26,065** | **453** | 2963253 | 0.879607647 | 1.737962785 | 1.528725357 |
| Nigeria | 0.00 | **25,694** | **590** | 206088236 | 0.012467475 | 2.296255935 | 0.028628514 |
| Israel | 11.00 | **25,547** | **320** | **9,197,590** | 0.277757543 | 1.252593259 | 0.347917226 |
| Ireland | 81.00 | **25,473** | **1,736** | 4937627 | 0.515895591 | 6.815059082 | 3.515858934 |
| Japan | 0.00 | **18,593** | **972** | 126474828 | 0.014700949 | 5.227773893 | 0.076853238 |
| Austria | 32.00 | **17,766** | **705** | 9006392 | 0.197259902 | 3.968253968 | 0.782777387 |
| Ghana | 0.00 | **17,741** | **112** | 31067780 | 0.057104177 | 0.631306014 | 0.03605021 |
| Moldova | 16.00 | **16,613** | **545** | 4033927 | 0.411831944 | 3.280563414 | 1.35104081 |
| Serbia | 6.00 | **14,564** | **277** | 8737206 | 0.166689443 | 1.901950014 | 0.317034988 |
| Algeria | 7.00 | **13,907** | **912** | 43845925 | 0.031717885 | 6.557848565 | 0.208001086 |
| S. Korea | 0.00 | **12,850** | **282** | 51269286 | 0.025063739 | 2.194552529 | 0.055003692 |
| Denmark | 33.00 | **12,768** | **605** | 5792222 | 0.22043354 | 4.738408521 | 1.044504164 |
| Morocco | 3.50 | **12,596** | **228** | 36909144 | 0.034127045 | 1.810098444 | 0.06177331 |
| Cameroon | 0.00 | **12,592** | **313** | 26539183 | 0.047446826 | 2.48570521 | 0.117938823 |
| Sudan | 2.00 | **9,257** | **572** | 43839762 | 0.021115534 | 6.179107702 | 0.130475161 |
| Norway | 32.00 | **8,887** | **250** | 5421191 | 0.163930767 | 2.813097783 | 0.461153278 |
| Malaysia | 0.00 | **8,639** | **121** | 32364451 | 0.026692867 | 1.400625072 | 0.037386699 |
| Finland | 3.50 | **7,214** | **328** | 5540737 | 0.130199286 | 4.546714721 | 0.591979009 |
| DRC | 0.00 | **7,122** | **175** | 89525683 | 0.007955259 | 2.457174951 | 0.019547463 |
| Senegal | 0.00 | **6,793** | **112** | 16739117 | 0.040581591 | 1.648756072 | 0.066909145 |
| El Salvador | 40.00 | **6,736** | **182** | 6486212 | 0.103851061 | 2.701900238 | 0.280595207 |
| Kenya | 0.00 | **6,366** | **148** | 53761146 | 0.011841265 | 2.32485077 | 0.027529175 |
| Ethiopia | 0.00 | **5,846** | **103** | 114934989 | 0.005086354 | 1.761888471 | 0.008961588 |
| Venezuela | 40.00 | **5,832** | **51** | 28435609 | 0.020509496 | 0.874485597 | 0.017935259 |
| Guinea | 0.00 | **5,391** | **33** | 13128762 | 0.041062516 | 0.61213133 | 0.025135653 |
| Bulgaria | 11.00 | **4,989** | **230** | 6948112 | 0.071803678 | 4.610142313 | 0.331025176 |
| Bosnia and Herzegovina | 2.00 | **4,453** | **186** | 3280702 | 0.135733145 | 4.176959353 | 0.566951829 |
| Hungary | 18.50 | **4,157** | **585** | 9660254 | 0.043031995 | 14.07264854 | 0.605574139 |
| Greece | 15.50 | **3,409** | **192** | 10422794 | 0.032707161 | 5.632150191 | 0.184211642 |
| Thailand | 0.00 | **3,173** | **58** | 69800238 | 0.00454583 | 1.827923101 | 0.008309427 |
| Somalia | 0.00 | **2,924** | **90** | 15888015 | 0.018403809 | 3.077975376 | 0.056646472 |
| Croatia | 8.50 | **2,777** | **107** | 4105122 | 0.067647198 | 3.853078862 | 0.260649988 |
| Palestine | 8.40 | **2,708** | **8** | 5100320 | 0.053094708 | 0.295420975 | 0.01568529 |
| Albania | 16.00 | **2,535** | **62** | 2877787 | 0.088088521 | 2.445759369 | 0.215443325 |
| Cuba | 40.00 | **2,341** | **86** | 11326595 | 0.020668171 | 3.673643742 | 0.075927496 |
| Mali | 0.00 | **2,181** | **116** | 20243694 | 0.010773725 | 5.318661165 | 0.057301795 |
| Sri Lanka | 0.00 | **2,047** | **11** | 21413307 | 0.009559476 | 0.537371764 | 0.005136993 |
| Slovakia | 14.50 | **1,687** | **28** | 5459649 | 0.030899422 | 1.659751037 | 0.051285348 |
| Zambia | 0.00 | **1,594** | **24** | 18377879 | 0.008673471 | 1.505646173 | 0.013059178 |
| Tunisia | 0.33 | **1,174** | **50** | 11818307 | 0.009933741 | 4.258943782 | 0.042307244 |
| Jordan | 17.80 | **1,132** | **9** | 10202906 | 0.011094878 | 0.795053004 | 0.008821016 |
| Niger | 0.00 | **1,075** | **67** | 24192310 | 0.004443561 | 6.23255814 | 0.027694751 |
| Cyprus | 9.50 | **998** | **19** | 1207351 | 0.082660303 | 1.903807615 | 0.157369315 |
| Burkina Faso | 0.00 | **962** | **53** | 20896692 | 0.004603599 | 5.509355509 | 0.025362866 |
| Georgia | 9.00 | **931** | **15** | 3989139 | 0.02333837 | 1.611170784 | 0.037602099 |
| Uganda | 0.00 | **893** |  | 45721113 | 0.001953146 | 0 | 0 |
| Chad | 0.00 | **866** | **74** | 16420123 | 0.005274017 | 8.545034642 | 0.045066654 |
| Libya | 0.00 | **824** | **24** | 6870905 | 0.011992598 | 2.912621359 | 0.034929896 |
| Jamaica | 40.00 | **702** | **10** | 2961174 | 0.023706814 | 1.424501425 | 0.03377039 |
| Zimbabwe | 0.00 | **591** | **7** | 14861921 | 0.003976606 | 1.184433164 | 0.004710024 |
| Tanzania | 0.00 | **509** | **21** | 59713693 | 0.000852401 | 4.125736739 | 0.003516781 |
| Vietnam | 0.00 | **355** |  | 97337022 | 0.000364712 | 0 | 0 |
| Myanmar | 0.00 | **299** | **6** | 54409575 | 0.000549536 | 2.006688963 | 0.001102747 |
| Syria | 15.00 | **279** | **9** | 17496498 | 0.001594605 | 3.225806452 | 0.005143887 |
| Mongolia | 0.00 | **220** |  | 3278003 | 0.006711403 | 0 | 0 |
| Cambodia | 0.00 | **141** |  | 16717979 | 0.000843403 | 0 | 0 |
